# Sustainable catalysis

**DOI:** 10.3762/bjoc.12.167

**Published:** 2016-08-08

**Authors:** Nicholas J Turner

**Affiliations:** 1 School of Chemistry & Manchester Institute of Biotechnology, The University of Manchester, 131 Princess Street, M1 7DN, Manchester, United Kingdom

**Keywords:** green chemistry, sustainable catalysis

This focused collection of papers is devoted to recent developments in ‘green chemistry’, especially the applications of catalytic methods, involving both chemo- and biocatalysis. The development of catalytic processes is an important current theme in organic chemistry, since it aims to reduce the environmental impact of the industrial synthesis of chemicals and polymers by replacing stoichiometric reagents with catalysts and also exchanging harmful organic solvents with less detrimental alternatives. Green processes can also result in lower costs of goods and starting materials as well as in the use of less harmful and toxic reagents, which delivers benefits in terms of safety and disposal of waste byproducts. Recently, many of these aspects of green chemistry have been the focus of the CHEM21 project (http://www.chem21.eu), funded by the Innovative Medicines Initiative, which is supported by the European Federation of Pharmaceutical Industries Association (EFPIA). CHEM21 is a large multi-group consortium organized into six different work-packages spanning various elements of green chemistry including biocatalysis, synthetic biology and non-precious metal catalysis. In addition, there are also work packages devoted to defining current and future targets for green chemistry as well as developing metrics-based assessment protocols and also training packages aimed at embedding the principles of green chemistry within the thinking of future scientists. It is hoped that the outputs from CHEM21, including some of the papers presented here, together with other similar initiatives, will contribute to the overall effort underway globally aimed at transforming the chemical industry into a more sustainable and environmentally operation.

Nicholas Turner

Manchester, July 2016

